# Supplementary material for: Identification of MHCII variants associated with chlamydial disease in the koala (Phascolarctos cinereus)
Source: PeerJ. 2014 Jun 19;2:e443. doi: 10.7717/peerj.443 (PMC4081129; doi:10.7717/peerj.443)
Supplement: Supplemental Information — A shows Fisher’s exact test comparing proportion of MHCII variant-positive c-hsp60 antibody seropositive and seronegative koalas. Supplemental Information B tabulates mean c-hsp60 antibody levels (logSU) associated with specific MHCII variants, which are also presented graphically in Fig. 2. [file peerj-02-443-s001.docx]

**Supplementary material**

**Supplementary material A**. Fisher’s exact test comparing proportion of variant-positive c-hsp60 seropositive koalas to seronegative koalas.

| Variant | Proportion c-hsp60 seronegative (%) | Proportion c-hsp60 seropositive (%) | Fisher’s exact p-value |
| --- | --- | --- | --- |
| DAB*10 | 49 / 60 (81.7%) | 26 / 33 (78.8%) | 0.787 |
| DAB*15 | 13 / 60 (21.7%) | 3 / 33 (9.1%) | 0.158 |
| DAB*22 | 14 / 60 (23.3%) | 8 / 33 (24.2%) | 1.00 |
| DAB*24 | 16 / 60 (26.7%) | 8 / 33 (24.2%) | 1.00 |
| DBB*01 | 5 / 60 (8.3%) | 6 / 33 (18.2%) | 0.189 |
| DBB*02 | 53 / 60 (88.3%) | 27 / 33 (81.9%) | 0.533 |
| DBB*03 | 21 / 60 (35.0%) | 12 / 33 (36.4%) | 1.00 |
| DBB*04 | 7 / 60 (11.7%) | 7 / 33 (21.2%) | 0.238 |

**Supplementary material B**. Association of DAB and DBB variants with chlamydial heat-shock protein 60 antibodies identified by General linear regression.

| Variant | Variant (+) | Variant (-) | p-value |
| --- | --- | --- | --- |
| DAB*10 |  |  |  |
| Mean + s.e. logSU | 0.64 ± 0.19 | 0.95 ± 0.44 | 0.552 |
| DAB*15 |  |  |  |
| Mean + s.e. logSU | 0.43 ± 0.45 | 0.76 ± 0.18 | 0.523 |
| DAB*22 |  |  |  |
| Mean + s.e. logSU | 1.38 ± 0.94 | 0.49 ± 0.33 | 0.472 |
| DAB*24 |  |  |  |
| Mean + s.e. logSU | -0.10 ± 0.94 | 0.98 ± 0.36 | 0.391 |
| DBB*01 |  |  |  |
| Mean + s.e. logSU | 1.50 ± 0.47 | 0.60 ± 0.16 | 0.073 |
| DBB*02 |  |  |  |
| Mean + s.e. logSU | 0.77 ± 0.17 | 0.31 ± 0.47 | 0.379 |
| DBB*03 |  |  |  |
| Mean + s.e. logSU | 0.49± 0.28 | 0.81 ± 0.20 | 0.380 |
| DBB*04 |  |  |  |
| Mean + s.e. logSU | 1.74 ± 0.41 | 0.52 ± 0.17 | **0.008** |

Significant p-values < 0.05 are in bold
